# The Influence of Spraying Strategy on the Dynamic Response of Polyurea-Coated Metal Plates to Localized Air Blast Loading: Experimental Investigations

**DOI:** 10.3390/polym11111888

**Published:** 2019-11-15

**Authors:** Yongqing Li, Changhai Chen, Hailiang Hou, Yuansheng Cheng, Haopeng Gao, Pan Zhang, Ting Liu

**Affiliations:** 1 College of Naval Architecture and Ocean Engineering, Naval University of Engineering, Wuhan 430033, China; 2 School of Naval Architecture and Ocean Engineering, Huazhong University of Science and Technology, Wuhan 430074, China; 3 Unit 91439 of PLA, Dalian 116041, China

**Keywords:** dynamic response, air blast, polyurea, steel, spraying strategy

## Abstract

Polyurea has attracted considerable attention owing to its potential applications in protective fields to improve the resistant performance of structures subjected to damage loads resulting from intentional or accidental explosions. However, different spraying strategies of polyurea may lead to significant differences in overall resistance performance of polyurea-coated structures, and the underlying mechanisms have not been clear until now. This study aims to elucidate the influence of spraying strategy, i.e., spraying area, spraying thickness, and spraying interface condition, on the dynamic response of polyurea-coated steel plates under localized air blast loading. Three types of plates manufactured using different spraying strategies were adopted to evaluate their blast-resistant performance. The spraying strategies used were (i) whole-area spraying, (ii) partial-area spraying, and (iii) in-contact backing of polyurea on the rear surfaces of steel plates. In addition, the influence of spraying thickness of polyurea for whole-area sprayed plates was evaluated. The energy absorbing mechanisms of polyurea backing layers were highlighted. The energy absorption of plates was quantitatively analyzed. The results show that the air blast resistances of whole-area sprayed and in-contact backed plates are both superior to, whereas that of partial-area sprayed plates is inferior to, bare steel counterparts. A suitable spraying thickness of polyurea can significantly reduce the damage of the front steel layer, whereas excessive spraying thickness decreases the overall air blast resistance of plates. The polyurea backing layer exhibits favorable performance in absorbing energy under a whole-area spraying condition. This study provides useful guidance for the design of polyurea-coated metal plates in engineering applications.

## 1. Introduction

Polymeric elastomers, like polyurea and polyurethane, provide an effective method for retrofitting, repair, reinforcement, and protection of building/civil infrastructures [1,2] and marine/aerospace structures [3,4,5], mainly owing to their high elongation, favorable abrasive properties, and preferable shock-mitigation performance. Under high rate conditions, polyurethane and polyurea have similar rate-dependent behaviors and both show strong hysteresis and phase transition to a glassy state [6,7] due to the breakage of urethane/urea linkages [8]. Even at intermediate strain rates, polyurethane exhibits viscoelasticity [7]. In terms of shock-mitigation, polyurethane/polyurea are commonly used as interlayers or claddings in sandwich structures under blast/impulsive loads. Bahei-El-Din and Dvorak [9] have investigated the dynamic behavior of polyurethane/polyurea-reinforced sandwich plates subjected to blast loads and indicated that the introduction of polyurethane/polyurea interlayers leads to a pronounced reduction of core compression and overall deflection. Zhang et al. [10] have carried out an experimental study on the deflection response of sandwich panels with a polyurethane core subjected to air blast loads, and their results revealed that filling a polyurethane core was an effective method of reducing the deflections of face sheets. Jamil et al. [11] have experimentally and numerically studied the blast response of aluminum/polyurethane sandwich plates. They have pointed out that the increase in the thickness of the polyurethane core could increase the overall resistant performance of the structure. When employed as a cladding, polyurethane can also mitigate blast hazards and thus increase the survivability of structures. Mostafa et al. [12] have proposed a lightweight protection technique to enhance the resistance of explosive-transporting facilities by surface-mounting polyurethane foam on plates, and have demonstrated the effectiveness of this technique. Codina et al. [13] have given an alternative to reduce the damage of reinforced concrete (RC) members subjected to close-in explosions, suggesting that the reinforcement of polyurethane results in excellent damage reduction. Ousji et al. [14] have presented an experimental and analytical approach to predict the air blast response of sacrificial polyurethane cladding by taking account of fluid-structure interaction. Low density of cladding was found to result in low plateau stress. Compared with polyurethane, polyurea is commonly synthesized by a co-polymerization reaction without catalysts. Additionally, polyurea has a much simpler spraying process and a significantly shorter curing time than polyurethane. In terms of its material properties, polyurea has more excellent characteristics. Hence, the application of polyurea is more widespread.

Polyurea, as a polymeric elastomer, has attractive features which reduce the damage of structures which results from severe shock loading, due to its special mechanical behavior. Some experiments have been conducted to study polyurea’s mechanical behavior at different strain rates [15,16,17] in order to obtain a constitutive model [18,19,20,21]. Polyurea shows hyperelasticity at low strain rates, but when the strain rates are high, the viscoelasticity effect cannot be ignored. By considering this, Clifton et al. [22] have developed a quasilinear viscoelasticity model, and Bai et al. [23] have presented a hyper-viscoelastic model for the dynamic response of polyurea over a wide strain-rate range. Different dynamic responses of polyurea have been attributed to differences in loading condition, especially in loading rate. At low loading rates, polyurea exhibits a rubbery-like state with weak strain rates. As the loading rates increase, the state of polyurea changes from rubbery to glassy. At high loading rates, the responses of polyurea are strain-rate dependent with viscoelasticity. Moreover, other loading conditions, such as loading pressure, environmental temperature, and load type, also affect the dynamic response of polyurea. With regard to the effect of loading rate, Roland et al. [15] have experimentally investigated the mechanical behavior of polyurea at medium strain rates of up to 1000/s with the help of a new drop-weight tensile test instrument. The results showed that there was no indication of a transition from a rubbery state to a glassy state for polyurea in the medium strain-rate range. Guo et al. [17] and Miao et al. [24] have conducted compressive and tensile tests over wide ranges of strain rates on polyurea. Their results revealed that polyurea displayed a high rate-dependent behavior with the strain hardening effect and that the sensitivity of polyurea became pronounced as loading rates increased. Jajam et al. [25] have performed a series of tensile, fracture, and impact tests to study the response of interpenetration polymer networks (IPNs) with polyurethane as the ductile phase, with these tests indicating that loading rates greatly affect the fracture morphology of IPNs. Roland and Casalini [26] have explored the influence of hydrostatic pressure on the dynamic response of polyurea. It was found that hydrostatic pressure is important for mitigating structural damage from impact loading. The effects of temperature on the behavior of polyurea have mainly focused on the phase transition of polyurea [17,27]. Results obtained by Miao et al. [24] have further revealed that temperature mainly affects the dispersal of soft segments in the hard domain of polyurea. With regard to the effect of load type, it is generally agreed that the strain-hardening effect plays an important role in the response of polyurea under conventional shock loading. Nevertheless, under the loading of laser-induced high amplitude pulses, the dynamic response of polyurea is dominated by the resultant effect of high-amplitude shear waves and secondary longitudinal waves [28,29,30].

With respect to the energy absorption behavior of polyurea, differences also exist under different dynamic loading scenarios. When subjected to conventional blast/shock wave loads, strain deformation and phase transition dominate the energy-absorbing process of polyurea [31,32]. Under ballistic impact, the main pathways of energy absorption for polyurea are ductile/brittle fractures due to large-scale cracks [33,34,35,36]. In addition, the possible transition of polyurea between its rubbery and glassy states under high-velocity ballistic impact conditions also contributes, to a certain extent, to the energy absorption of polyurea [37]. When a polyurea thin film suffers a laser-generated shear wave loading, its energy absorption is mainly due to interfacial failure as well as shear failure due to high-intensity shear waves [29,30]. Nevertheless, further investigations by Jajam and Scottos [28] have shown that shear is the dominant energy-absorbing type for a polyurea thin film under a laser-induced dynamic mixed-mode condition.

Recently, polyurea has attracted much attention because of its benefits in improving the impact resistant performance of structures and as a main mitigation component in structures under blast and impact loadings. When used as a sandwich core or interlayer material, polyurea can largely mitigate blast loadings and absorb a large amount of impact energy [9,38]. Grujicic et al. [39] have conducted a dynamics computational analysis of polyurea suspension pads in helmets which indicated that polyurea pads could greatly reduce peak loading. Haris et al. [40] have conducted a series of experiments to investigate the mitigation performance of polyurea suspension pads. Their results revealed that the use of polyurea pads of 4 mm thickness could result in peak pressure and impulse of approximately 74% and 49%, respectively. Nevertheless, when polyurea is directly spray-cast onto a metal plate, its influence depends on its relative position. According to the available literature, when polyurea is sprayed on the back face of a metal plate, the overall performance of the plate improves. However, different and even contradictory viewpoints have been obtained for some studies when polyurea has been applied on the front face of a metal plate. Amini et al. [41,42] have investigated the effect of the relative position of polyurea on the dynamic response of steel plates subjected to impulsive loading. They found that polyurea can reduce the damage level of steel plates when sprayed on the back face of the steel plate, whereas the performance of steel plates deteriorates when polyurea is cast onto the front face of the steel plate. Samiee et al. [43] have numerically studied the influence of polyurea on the dynamic response of steel plates subjected to blast-like loads and have pointed out when the polyurea is sprayed on the back face of a steel plate the most effective blast-wave mitigation is gained. However, Rijensky and Rittel [44] have investigated the dynamic response of polyurea-coated aluminum plates under hydro-dynamic loading and have revealed that a better mitigation effect is acquired when polyurea is placed on the loading side. An experimental study by Dai et al. [45] has shown that a reduction in the deformation of steel plates subjected to underwater explosive loading can be gained in spite of the position of polyurea. Recently, Li et al. [46] have experimentally studied the effect of polyurea coating on the dynamic response of aluminum plates subjected to underwater shock wave loading. Their results showed that aluminum plates with polyurea sprayed on their front face had identical shock resistance as those which had polyurea sprayed on their back face, and that both types of plates displayed superior shock resistance to bare steel counterparts.

The abovementioned contradictory viewpoints about polyurea’s ability to resist blast/impulsive loads can be mainly attributed to differences in load intensity, load pattern, and polyurea’s material properties. Under low-intensity shock wave loading, polyurea applied on the front surface of a metallic plate can mitigate shock waves, whereas it will transmit more impact energy to the plate under high-intensity shock wave loading. When subjected to hydrodynamic loadings, polyurea sprayed on the front side of a metallic plate can reduce cavitation damage because of its pressure sensitivity. Moreover, its presence increases both the overall strain and material internal energy of the structure.

Spraying strategies of polyurea, such as spraying area, spraying thickness, and spraying interface condition, significantly affect the performance of polyurea-coated metal plates under intense impulsive loading. Some studies on this subject are available. However, the influence of spraying strategy on the dynamic response of polyurea-coated steel plates under localized air blast loading is still not clear. This study aims to elucidate the influence of spraying strategy on the dynamic response of polyurea-coated steel plates under localized air blast loading by conducting air blast experiments. Three types of polyurea-coated steel plates manufactured using different spraying strategies were adopted. Three stand-off distances, i.e., 50, 100, and 150 mm, were used to completely evaluate the influence of spraying strategy. The deformation/failure modes of plates were identified after testing by conducting post-mortem deformation analysis. A comparison of air-blast-resistant performance was performed. The energy absorbing mechanisms of polyurea backing layers were elucidated. A quantitative analysis of energy absorption of the plates after testing was conducted.

## 2. Experimental Procedure

### 2.1. Material Properties

The polyurea LINE XS-350 was employed in the present experiments and was provided by the LINE-X Protective Coating Company (Huntsville, AL, US). The polyurea used has exceptional mechanical performance and thus is very suitable for applications under extreme conditions, such as blast/explosive loadings, as a pure polyurea coating with high-performance physical properties. LINE XS-350 is a two-component elastomer spraying material, with its raw materials including isocyanate as component A and RESIN as component B. The main constituent of RESIN is diamine oligomer. There is no dilution required.

**Figure 1:**
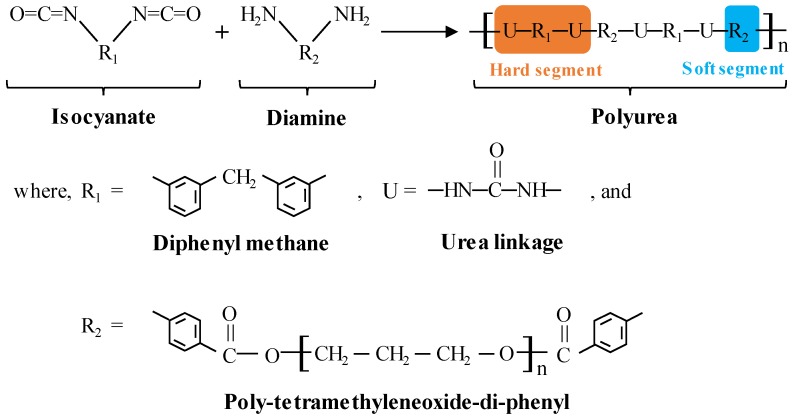
Schematic of the synthesis and chemical molecular structure of polyurea via the co-polymerization reaction of an isocyanate component and a synthetic resin blend.

The polyurea used is formed by the copolymerization reaction of isocyanate and diamine compounds, and its molecular structure is shown in Figure 1. Polyurea is a microphase-separated block polymer material composed of a hard segment and a soft segment. The hard segment contains a π-type stacked aromatic chain and two urea-linkages. The soft segment consists of a flexible aliphatic compound. During the synthesis process, hard segments are evenly distributed in the soft segment matrix at room temperature, leading to interconnected network microstructures. The strength of polyurea is mainly related to hard segments and the elongation of polyurea is relevant to soft segments.

**Figure 2:**
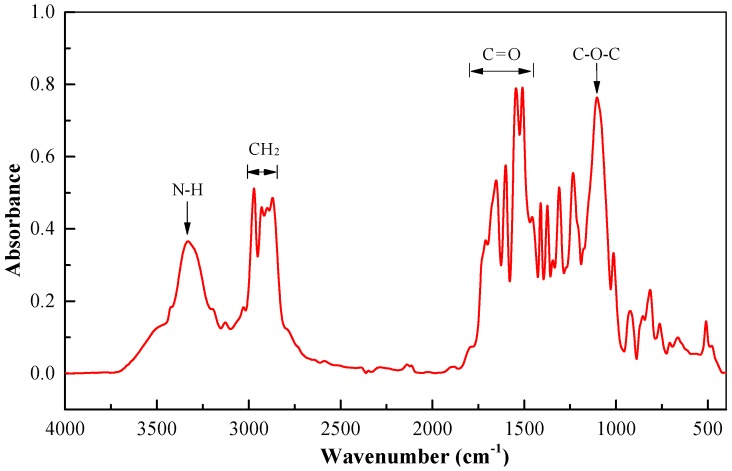
FT-IR spectroscopy of LINE-X 350 polyurea with typical peaks.

Fourier-transform infrared (Bruker Co., Karlsruhe, Germany) testing of the LINE-X 350 polyurea material was performed. As shown in Figure 2, an absorption peak in the vicinity of 3320 cm^−1^ was associated with the stretching vibration of N–H bonds. Absorption peaks in the approximate range of 2870 cm^−1^ to 2971 cm^−1^ were induced by the symmetric stretching vibration of CH_2_ bonds. The carbonyl group containing a C=O bond produced absorption peaks in the spectral range of 1450 cm^−1^ to 1800 cm^−1^ which were mainly due to the high content of C=O bonds in polyurea. Absorption peaks in the vicinity of 1310 cm^−1^ resulted from the stretching vibrations of N–H and C–H bonds on aromatic rings. Absorption peaks in the vicinity of 1082 cm^−1^ and 1100 cm^−1^ were caused by the symmetric and asymmetric stretching vibrations of C–O–C bonds, respectively, which had a certain extent of superposition. The in-plane and out-of-plane bending vibrations of C–H bonds on benzene rings produced absorption peaks near 1018 cm^−1^ and 920 cm^−1^, respectively. Moreover, the absorption peaks in the vicinity of 770 cm^−1^ were mainly induced by the out-of-plane bending vibration of O=C–O bonds.

**Figure 3:**
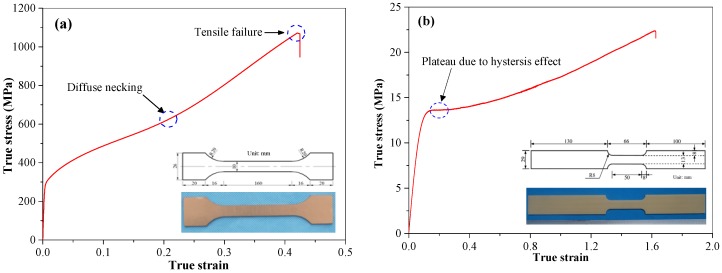
Quasi-static true stress–strain curves of (**a**) 304 stainless steel and (**b**) LINE XS-350 specimens.

304 stainless steel, whose material properties are listed in Table 1, was utilized to manufacture the polyurea-coated steel plates as the front steel layers; the corresponding quasi-static stress–strain curve is shown in Figure 3a.

The material properties of polyurea LINE XS-350 are listed in Table 1, and the corresponding stress–strain curve obtained through quasi-static tensile tests is depicted in Figure 3b.

### 2.2. Specimen Design and Manufacture

With different spraying strategies, three types of polyurea-coated steel plates, i.e., SPW, SPP, and SPC, were designed, as shown in Table 2. SPW denotes polyurea which is sprayed on the whole area of the back side of the steel plate. SPP denotes polyurea which is sprayed onto a partial area (central area) of the back side of the steel plate. SPC denotes polyurea in which the polyurea backing layer is in contact with the rear surface of the steel plate. For comparison, bare steel plates were used and denoted BS.

**Figure 4:**
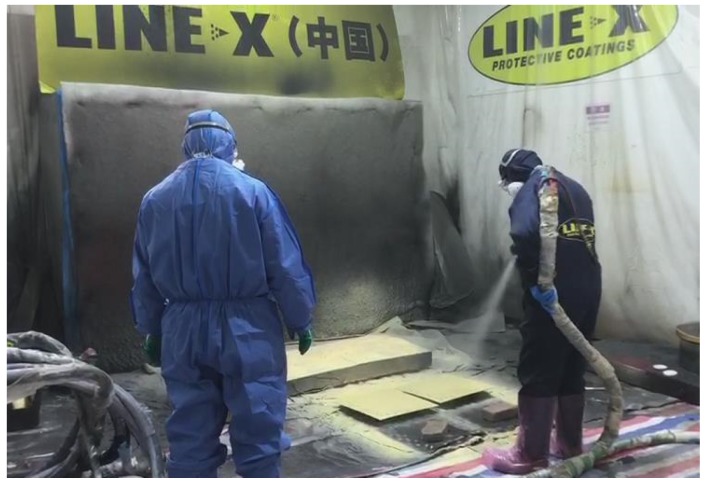
Polyurea spraying process using the spray polyurea elastomer (SPUA) technique.

The SPW plates were fabricated by the direct spraying of polyurea on the whole area of the back surface of a steel plate by the spray polyurea elastomer (SPUA) technique, as shown in Figure 4. Before the spraying process, the two types of raw materials used for LINE XS-350, i.e., the A and B components, were injected into a special device with a volume ratio of 1:1. Then, the resulting composition was stirred together until the two components had mixed uniformly in the temperature range 21–32 °C. Just prior to spraying, the mixture was heated to 60–70 °C in order to achieve the best mixing and co-polymeric effects. During the spraying process, the mixture was pressurized to 13.8–17.2 MPa and then immediately sprayed on the back surface of the steel plate using a special high-pressure ejecting device. After spraying, the polyurea coating cured within 3 to 5 s at room temperature.

The spraying area of the SPW plates was 452 mm (*L*, length) × 440 mm (*B*, width). Two spraying thicknesses of polyurea coatings, i.e., 3.1 mm and 6.6 mm, were used for the SPW plates to investigate the influence of the spraying thickness of polyurea.

The SPP plates were manufactured in a similar manner as the SPW plates, but for these plates the spraying area was different. For the SPP plates, polyurea was directly sprayed on a partial area (central area) of the back face of the steel plates with dimensions of 300 mm (*L*_e_, length) × 288 mm (*B*_e_, width), with a 3.1 mm spraying thickness of polyurea. For the SPW and SPP plates, the surfaces of the steel plates were degreased, roughly polished, sandblasted, and cleaned just prior to spraying. Within the SPW and SPP plates, there were strong bonding interfaces between the front steel layers and the polyurea backing layers. The interface bonding conditions in the SPW and SPP plates represent those plates with strong bonding strength.

The SPC plates were manufactured in two steps, i.e., via direct spraying of polyurea onto the whole back surface of the steel plate with dimensions of 452 mm × 440 mm, and then separation of the polyurea layer from the steel plate. The spraying thicknesses of polyurea in all the SPC plates were 3.1 mm. In the SPC plates, the interfaces between the front steel layers and the polyurea backing layers were in contact, and were adopted to mimic an extreme interface condition with zero bonding strength, representing interface conditions with relatively weak bonding strength.

For the as-received 304 stainless steel plates before processing, their surface roughness grades were in the range Sa0.1–Sa1.0, i.e., the surface roughness conditions were in the range 0.1–1.0 μm. After the sandblasting process, the surface roughness grades of the steel plates were approximately Sa2.5.

### 2.3. Experimental Set-Up

**Figure 5:**
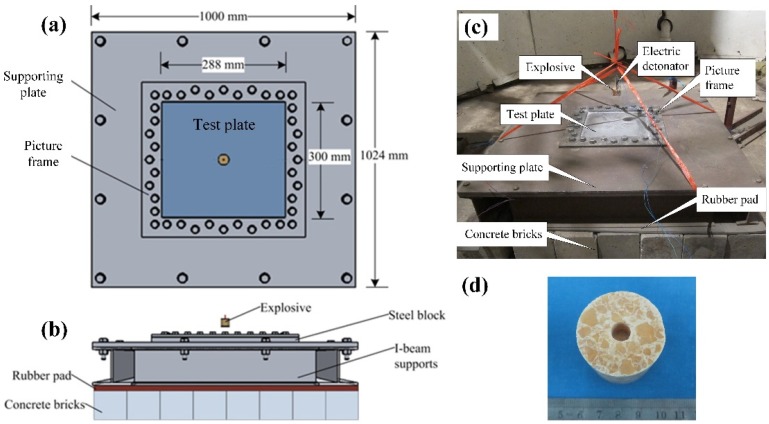
(**a**) Top view and (**b**) side view of a schematic of the clamping and supporting structures; (**c**) a photograph of the test set-up just prior to detonation; (**d**) picture of the cylindrical TNT charge.

All the air blast experiments were implemented in an explosion chamber with a 5 m inner diameter and 7.5 m height [10]. The details of the testing system are provided in the literature [47]. A photograph of a typical test set-up just prior to detonation is shown in Figure 5. The exposed region in the testing system was 300 mm (*L*_e_) × 288 mm (*B*_e_). The information of the mass of the explosive and stand-off distance in each test case is presented in Table 3, in which *δ*_s_ is the maximum permanent deflection and *M*_s_ denotes the failure mode of the front steel layer in polyurea-coated steel plates. The stand-off distances (*SoD*s) are the vertical distances between the mass center of the explosive and the front surface of the tested plates.

## 3. Experimental Results

Table 3 shows the experimental results of the polyurea-coated steel plates, where *h*_s_ and *h*_p_ are the thicknesses of the front steel layer and polyurea backing layer, respectively; *W*_t_ and *ρ*_A_ are the total mass and total areal density of the tested plates, respectively; *SoD* represents the stand-off distance of the explosive and *W*_e_ is the mass of the TNT charge; and *δ*_s_ and *M*_s_ denote the maximum permanent deflection and deformation/failure modes of the front steel layers, respectively.

In addition, bare steel plates made of 304 stainless steel with 1.80 mm thicknesses were employed here as a baseline. Note that the results of plates BS-1, BS-2, and BS-3 were taken from Ref. [47]. For a clear comparison, the related information of the three plates is presented in Table 4.

The experimental results have been divided into four groups based on the following observations: (i) permanent deflections of steel layers/plates, (ii) observations about deformation/failure modes, (iii) crevasse/hole size of front steel layers, and (iv) spalling area of polyurea backing layers. The details are illuminated in Section 3.1, Section 3.2, Section 3.3, and Section 3.4.

### 3.1. Permanent Deflections of Steel Layers

The maximum permanent deflections (*δ*_s_) of front steel layers under Mode I in the polyurea-coated steel plates were measured after testing and are included in Table 3. A comparison of air blast resistance in terms of maximum permanent deflections was conducted as mentioned in Section 4.

### 3.2. Deformation and Failure Modes

#### 3.2.1. Front Steel Layers

**Figure 6:**
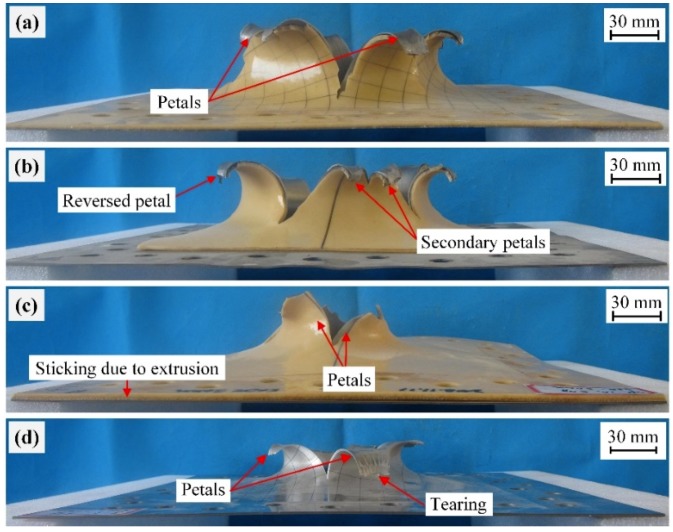
Photographs of the damaged plates (**a**) SPW-1, (**b**) SPP-1, (**c**) SPC-1, and (**d**) BS-1 (from Ref. [47]) tested at a stand-off distance of 50 mm.

Figure 6 shows the side views of the damaged front steel layers for SPW, SPP, SPC, and BS plates when tested at a stand-off distance of 50 mm. The front steel layers for all plates, SPW-1, SPP-1, and SPC-1, exhibited petalling failure at the central regions, in a similar fashion to plate BS-1, as shown in Figure 6. Differences existed in the petalling crevasse size; this is analyzed in Section 3.3. Hence, the front steel layers in the polyurea-coated steel plates all failed due to a petalling mechanism under intense air blast loading, similarly to the bare steel counterparts.

**Figure 7:**
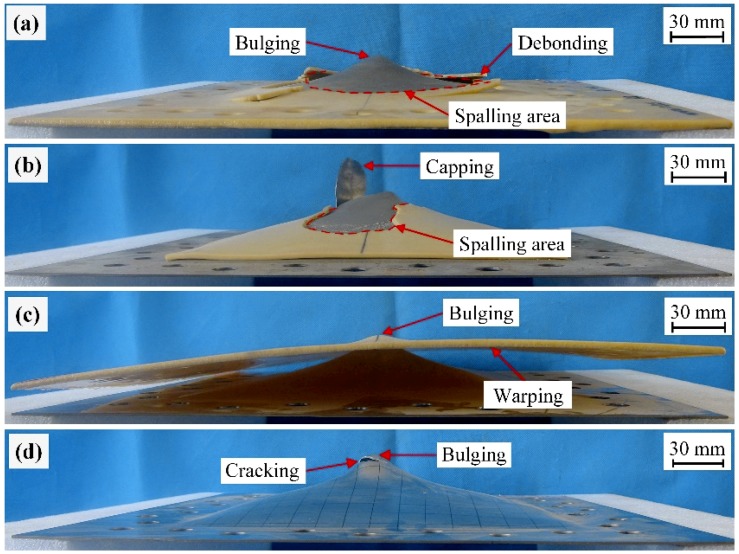
Photographs of the damaged plates (**a**) SPW-2, (**b**) SPP-2, (**c**) SPC-2, and (**d**) BS-2 (from Ref. [47]) tested at a stand-off distance of 100 mm.

When tested at a stand-off distance of 100 mm, the front steel layers for plates SPW-2 and SPC-2 experienced large plastic deformation, whereas that for plate SPP-2 failed by Mode IIc, as shown in Figure 7. This indicates that polyurea-coated steel plates with partial-area spraying have inferior performance compared to those with whole-area spraying and in-contact backing. Further comparison with plate BS-2, as shown in Figure 7, indicates that the damage extent of plate SPP-2 was higher than that of plate BS-2. The difference in the failure mode of the front steel layers can be attributed to the two following factors: (1) a partial-area sprayed polyurea layer slightly benefits the necking retardation of the front steel layer due to the lack of boundary confinement and (2) a partial-area sprayed polyurea backing layer is prone to move rather than stretch together with the front steel layer, and thus its energy absorbing capability cannot be sufficiently utilized.

**Figure 8:**
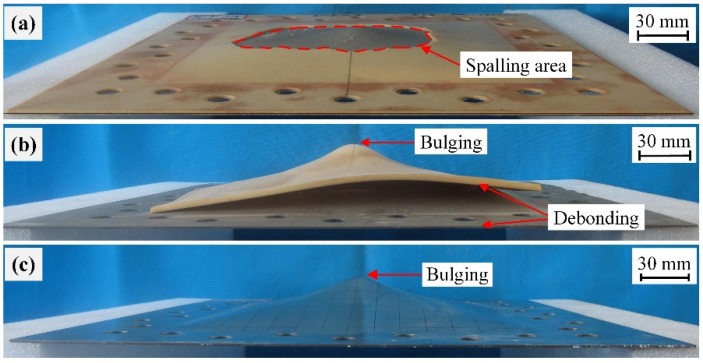
Photographs of the damaged plates (**a**) SPW-3, (**b**) SPP-3, and (**c**) BS-3 (from Ref. [47]) tested at a stand-off distance of 150 mm.

Further increasing the stand-off distance to 150 mm led to the deformation/failure mode of the front steel layers in plates SPW-3 and SPP-3 changed to Mode I, similarly to plate BS-3, as shown in Figure 8. At a 150 mm stand-off distance, it can be easily inferred that the front steel layer in an SPC plate should also exhibit Mode I deformation given the result obtained with a 100 mm stand-off distance. Hence, the front steel layers in SPW, SPP, and SPC plates all exhibited Mode I deformation when the intensity of the air blasts was relatively low.

#### 3.2.2. Polyurea Backing Layers

Figure 6 shows the failure modes of polyurea backing layers in plates SPW-1, SPP-1, and SPC-1, which were all petalling; no debonding was visually detected when testing at a stand-off distance of 50 mm. Nevertheless, sticking of the polyurea backing layer was visually detected for plate SPC-1, mainly because of the extrusion action exerted by the front steel layer at intense air blast loads.

When tested at a stand-off distance of 100 mm, the failure modes of the polyurea backing layers were different. As shown in Figure 7, the polyurea backing layers in plates SPW-2 and SPP-2 both exhibited spalling and that in plate SPC-2 exhibited stretching deformation. Moreover, premature polyurea fragments were visually detected in plate SPW-2. After testing, polyurea fragments were collected for plate SPW-2, indicating that the fragmentation of the polyurea backing layer might have occurred. As shown in Figure 7c, the polyurea backing layer of plate SPC-2 was deformed by large stretching. This can be mainly attributed to the combined effect of boundary confinement and a lack of interface bonding. Moreover, warped edges were formed. This can be attributed to the in-contact interface in plate SPC-2 which made the polyurea backing layer collide and then extrude by the front steel layer in the process of deformation. No spalling was produced on the polyurea backing layer of plate SPC-2, a significantly different result from those backing layers in plates SPW-2 and SPP-2.

As the stand-off distance increased to 150 mm, spalling was still produced on the polyurea backing layer in plate SPW-3, whereas the polyurea backing layer in plate SPP-3 failed by intact debonding together with a centrally localized bulging deformation, as shown in Figure 8a,b. This difference is mainly due to the fact that the polyurea backing layer in a whole-area sprayed polyurea-coated steel plate has confined boundaries, while the boundaries of a polyurea backing layer in a partial-area sprayed plate are free. Hence, the polyurea backing layer in a partial-area sprayed plate tends to move together with the front steel layer rather than stretch and then produce spalling due to its free boundaries. It can be inferred that the polyurea backing layer in SPC plates exhibited stretching deformation at a stand-off distance of 150 mm.

### 3.3. Comparison of Crevasse/Hole Sizes

In order to compare the damage levels of the front steel layers which experienced petalling or Mode IIc, the sizes of crevasses or holes were measured. Table 5 shows the diameters (*d*_c_) of the equivalent circulars with comparative areas to measured crevasse/hole.

At the stand-off distance of 50 mm, the front steel layer of plate SPW-1 had 21.1% and that of plate SPP-1 had 24.5% larger petalling crevasse diameters than plate BS-1. This is mainly due to the fact that the thicknesses of the front steel layers for plates SPW-1 and SPP-1 were smaller than that of the front steel layer for plate BS-1. A steel layer with a relatively thin thickness would be damaged more severely in the form of a larger petalling crevasse under the same blast loading. However, the front steel layer of plate SPC-1 showed a 38.0% smaller crevasse diameter than plate BS-1; this phenomenon can be mainly attributed to the in-contact interface, which makes the energy absorbing capability of the polyurea backing layer in an SPC plate to be sufficiently utilized through stretching deformation. Moreover, the collision and friction between the layers in SPC plates would contribute to absorb kinetic energy to a certain extent.

With the same thickness and at the same stand-off distance of 50 mm, the diameter of the front steel layer with a capping hole in plate SPW-4 was 79.9% smaller than that of plate BS-1 with a petalling crevasse, as listed in Table 5. This is mainly due to the extra back-face sprayed polyurea coating which had two benefits, i.e., necking retardation of the front steel layer and its energy absorption. Hence, an extra rear-side sprayed polyurea coating could significantly reduce the damage of a steel plate. The front steel layer of plate SPP-2 exhibited a 41.3% larger hole size than the front steel layer in plate SPW-4, despite the fact that plate SPW-4 was tested at a closer stand-off distance; this is mainly due to the free boundaries in SPP plates.

### 3.4. Measurements of Spalling Area

Because the polyurea backing layers failed mainly due to spalling when tested at stand-off distances of 100 mm and 150 mm, the spalling area (*A*_s_) was measured and has been listed in Table 6. It can be observed that the spalling area in the polyurea backing layers increased as the stand-off distance decreased, and, in other words, spalling increased with increasing intensity of air blast loading. Under the same stand-off distance condition, the thicker the polyurea backing layers in SPW plates, the smaller the spalling area. This is mainly because a thicker polyurea backing layer in an SPW plate absorbs relatively more kinetic energy through stretching deformation, leaving less kinetic energy for the polyurea backing layer to produce spalling with. Moreover, a thicker polyurea layer has a larger inertia, making the spalling more difficult to occur.

As listed in Table 6, the polyurea backing layer in plate SPP-2 had a 60.0% smaller spalling area than that in plate SPW-2 at the same stand-off distance of 100 mm. This is mainly due to the following two aspects: (i) The front steel layer of plate SPP-2 with Mode IIc failure mode absorbs more impact energy than that of plate SPW-2 with Mode I deformation mode, and (ii) the polyurea backing layer is inclined to move together with the front steel layer in plate SPP-2 rather than to produce a large stretching deformation, due to the lack of confinement of its boundaries.

## 4. Discussions

### 4.1. Influence of Spraying Area

**Figure 9:**
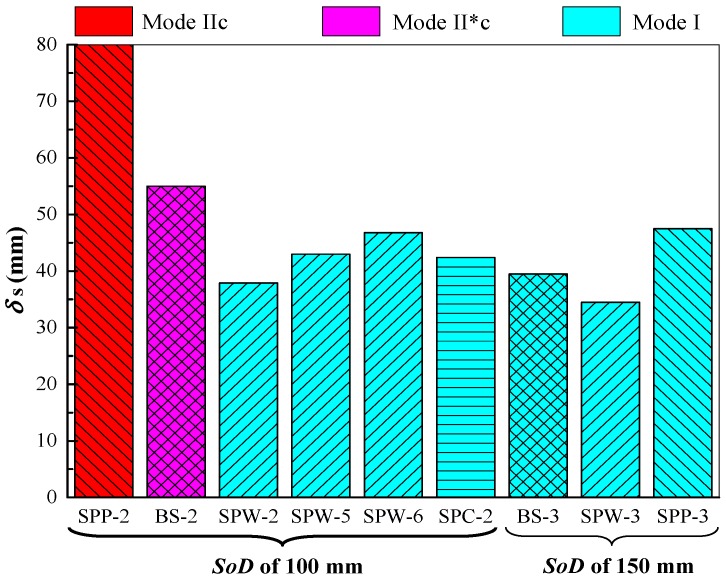
Influence of spraying strategy on the performance of polyurea-coated steel plates under air blasts.

Figure 9 shows a comparison of the performance of the tested plates when resisting air blast loading at 100 mm and 150 mm stand-off distances. In Figure 9, a pseudo higher value of deflection for damaged plate SPP-2 was used for the purpose of comparison. It can be seen from Figure 9 that the front steel layer in plate SPW-2 exhibited Mode I deformation, whereas that in plate BS-2 exhibited Mode II*c failure mode under the same 100 mm stand-off distance condition. Furthermore, the front steel layer in plate SPW-2 had a smaller maximum permanent deflection than bare steel plate BS-2. For plate SPP-2 tested at a stand-off distance of 100 mm, its front steel layer exhibited Mode IIc failure; plate SPP-2 was damaged more severely than plate BS-2. Moreover, at the identical stand-off distance of 150 mm, the front steel layer in plate SPP-3 showed larger maximum permanent deflection than the bare steel plate BS-3, as shown in Figure 9. Hence, the air-blast-resistant performance of SPP plates is inferior to that of bare steel counterparts. The lack of boundary confinement on the polyurea backing layer in an SPP plate is the main reason for this, as analyzed and presented above.

Thus, polyurea-coated steel plates with whole-area spraying of polyurea displayed superior performance, whereas those with partial-area spraying of polyurea showed inferior performance in comparison with their bare steel counterparts under the condition of identical total areal density. This conclusion is generally consistent with those obtained by Hui and Oskay [48] and Ackland et al. [49]. Nevertheless, the blast loads in Ref. [48] were created by a shock tube and simulated as uniform loads. Furthermore, the failure of the polyurea layer was not taken into account and attention was mostly paid to the deformation processes of polyurea-coated plates in both Refs. [48] and [49].

### 4.2. Influence of Spraying Thickness

Figure 9 shows that at the same stand-off distance of 100 mm, the front steel layers in plates SPW-5 and SPW-6 both had larger maximum permanent deflections than the front steel layer of plate SPW-2. Actually, the thickness of the polyurea backing layer in plate SPW-5 was equal to that in plate SPW-2, whereas that of the front steel layer of plate SPW-5 was higher than that of plate SPW-2, as listed in Table 3. Hence, the front steel layer in plate SPW-5 should have exhibited a smaller deflection than plate SPW-2; however, a contradictory result was obtained. Plate SPW-6 had an identical total areal density to plate SPW-2, but a thinner front steel layer than plate SPW-2. Thus, the front steel layer of plate SPW-6 should have exhibited a larger deflection than that of plate SPW-2, because the dominant energy absorbing component in a direct-spraying polyurea-coated steel plate is the front steel layer. This is consistent with the experimental result. By keeping the thickness of the steel layer the same as the 1.8 mm bare steel plate, the presence of an extra-sprayed 3.1 mm polyurea coating (plate SPW-4) significantly reduced the damage level of the steel layer in comparison with that of plate BS-1. This can be attributed to the necking retardation effect and energy absorption of the extra-sprayed polyurea backing layer in plate SPW-4. The front steel layers in plates SPW-5 and SPW-6 both experienced large deformation and exhibited Mode I deformation, but were different in terms of maximum permanent deflections.

**Figure 10:**
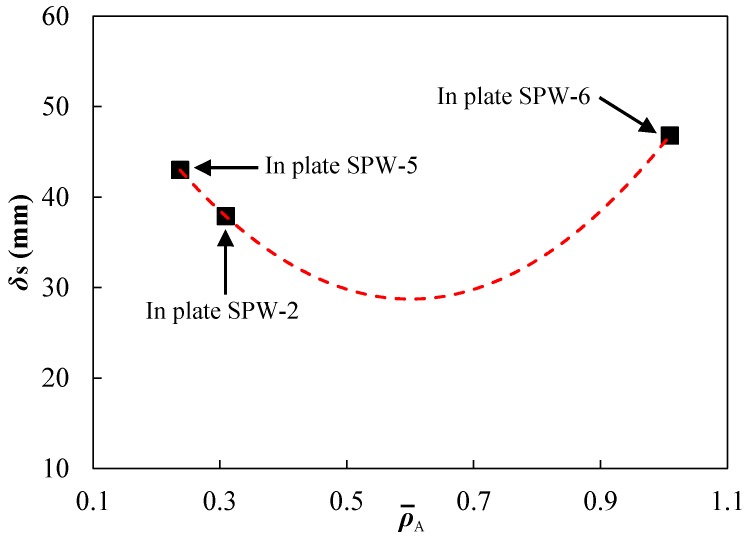
Influence of spraying thickness on the maximum permanent deflection (*δ*_s_) of the front steel layers in different polyurea-coated steel plates at a stand-off distance of 100 mm.

Figure 10 shows the influence of spraying thickness on the maximum permanent deflections of front steel layers, where ${\bar{\rho }}_{A}=\rho_{p}h_{p}/\left(\rho_{s}h_{s}\right)$ is the areal density ratio of the polyurea backing layer to the front steel layer, indicating the relative spraying thickness of the polyurea backing layer in a whole-area sprayed polyurea-coated steel plate. The maximum permanent deflection δ_s_ did not monotonously increase with increasing spraying thickness. The fitted curve (red dashed line in Figure 10) may not represent the variation trend, but it at least indicates that there is a matching relationship between the two layers in a whole-area sprayed plate. The two main reasons for this: (i) the relative stiffness of the polyurea backing layer to the front steel layer affects the compatible deformation between them during deformation, and (ii) the front steel layer has a higher energy absorbing capability.

When the spraying thickness, i.e., the thickness of the polyurea backing layer, is smaller than a threshold value, an increase in spraying thickness leads to a better compatible deformation between the layers. Meanwhile, the necking retardation effect of polyurea improves the energy absorption of the front steel layer. However, an excessive increase in the spraying thickness would lower the total energy absorption efficiency as the dominant energy absorbing component in a direct-spraying plate is the front steel layer. Understanding and determining the matching relationship between the spraying thickness and overall performance of polyurea-coated steel plates was deemed by us worth being investigated further. The conclusion made about the effect of polyurea layer thickness in this study was different to those gained by Amini et al. [42,50], Samiee et al. [43], Dai et al. [45], and Li et al. [46]. They have all indicated that increasing polyurea layer thickness always leads to the reduction of the deflection of steel layer, and have not considered the issues induced by an excessive increase in polyurea layer thickness. This can be mainly attributed to the lack of consideration of the spalling failure of the polyurea layer in Refs. [42,43,45,46,50]. Moreover, the thickness ratios of the polyurea layer to the metal layer in the above references were basically in a relatively small range. In fact, the conclusions in the above references regarding the influence of polyurea layer thickness agreed with that obtained in this study when the ratio of spraying thickness was relatively low, as shown in Figure 10.

### 4.3. Influence of Spraying Interface Condition

The front steel layer in plate SPC-2 exhibited Mode I deformation, whereas the failure mode of plate BS-2 was Mode II*c, at the same stand-off distance of 100 mm, as shown in Figure 9. Furthermore, the front steel layer of plate SPC-2 had a smaller maximum permanent deflection than plate BS-2. This is mainly because the polyurea backing layer in in-contact polyurea-coated plates, i.e., SPC plates, tends to produce a large stretching deformation, and its energy absorbing capability can be sufficiently utilized. This is the main reason that the air-blast-resistant performance of the in-contact polyurea-coated steel (SPC) plates is superior to that of bare steel plates with the same total areal density. As shown in Figure 9, the front steel layer of plate SPC-2 showed a higher maximum permanent deflection than the front steel layer of plate SPW-2. Thus, the blast-resistant performance of SPC plates is inferior to that of SPW plates in terms of deflection of the front steel layer. This is mainly because of the difference in the failure of the polyurea backing layers: the polyurea backing layer in an SPC plate experiences stretching deformation whereas that in an SPW plate is damaged by spalling. However, no spalling appeared on the polyurea backing layers of the SPC plates, and this would be beneficial to protecting personnel or vital equipment against possible secondary damage by fragments.

It is worth noting that Amini et al. [42,51] and Dai et al. [45] have also investigated the influence of interface bonding strength on the performance of polyurea-coated steel plates. However, they did not capture the failure mode variation of polyurea backing layers due to the change in interface bonding strength across a wide range. Moreover, more attention was given to the influence of interface bonding strength on the debonding response of the polyurea layer in Refs. [42,51]. No further investigation into the effect of interface bonding on the dynamic response of polyurea-coated steel plates was performed, and it was just pointed out that bonding strength may be one of the factors resulting in various experimental results in Ref. [45].

### 4.4. Energy Absorbing Mechanisms of Polyurea Backing Layers

The difference in energy absorbing mechanisms for different polyurea backing layers is in essence due to impedance mismatching in polyurea-coated steel plates from the perspectives of stress wave propagation and complex stress states during the blast-resistant procedure.

**Figure 11:**
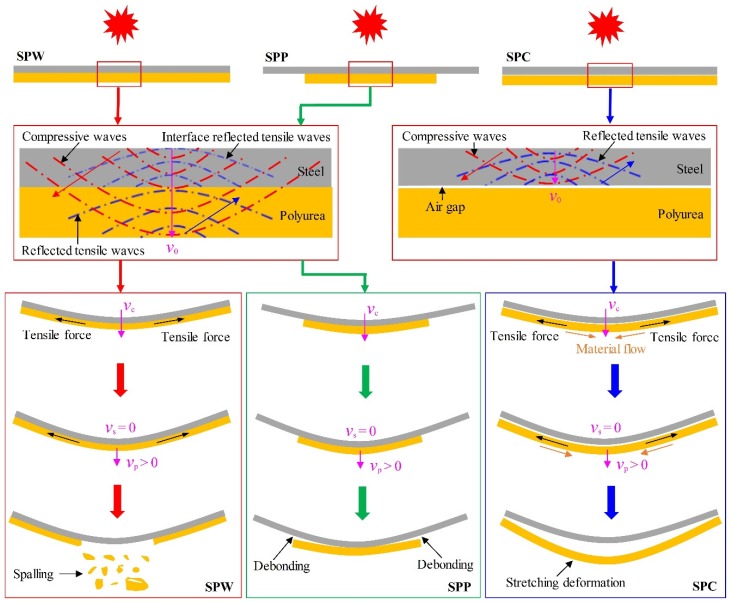
Schematic of stress wave propagation and complex stress states in polyurea backing layers during blast-resistant procedure for SPW, SPP, and SPC plates.

Figure 11 shows the stress wave propagation and complex stress states in polyurea backing layers during the blast-resistant procedure for SPW, SPP, and SPC plates, where *v*_0_, *v*_c_, *v*_s_, and *v*_p_ are the initial velocity of the plate or front steel layer, common velocity, and instantaneous velocities of the front steel layer and polyurea backing layer, respectively. Initial compressive wavefronts propagate in the through-thickness direction until the polyurea backing layer is reached. As these compressive waves reach the rearmost surface of the polyurea backing layer in the SPW and SPP plates, reflected tensile waves produce and propagate in the opposite direction, as shown in Figure 11. As the movement of the plate continues, the polyurea backing layer in the SPW plates undergoes deformation with the front steel layer and suffers the combined action of tensile force arising from its confined boundaries and extrusion by the front steel layer. When the intensity of air blast loading increases to a large enough extent, spalling is produced on the central region of the polyurea backing layer in SPW plates.

For partial-area sprayed polyurea-coated steel plates, i.e., SPP plates, the polyurea backing layers had free boundaries, and no tensile force acts, as shown in the middle part of Figure 11. Therefore, they tended to move along with the front steel layers rather than produce stretching deformation. The delamination initiates at the boundaries and progresses to the central region of the polyurea backing layer, leading to partial and then complete debonding as the intensity of air blasts further increases. Therefore, intact debonding from the boundaries proceeding towards the center would more likely occur for the polyurea backing layer in the polyurea-coated steel plates with partial-area spraying. When the intensity of air blast loading is relatively high, the polyurea backing layer does not have enough time to produce intact debonding, and, meanwhile, its central region has such high tensile stress that local spalling will occur. When the air blast loading is very intense, i.e., at a stand-off distance of 50 mm, central petalling will be produced for the polyurea backing layer.

For the SPC plates, the initial compressive waves just reached the steel–air interface and then reflected back. As the front steel layer moves forward, collision occurs between the polyurea backing layer and front steel layer and they then move together with a common velocity *v*_c_. The polyurea backing layer undergoes deformation with the front steel layer and suffers the combined action of tensile force arising from its confined boundaries and extrusion by the front steel layer. However, material flow occurs towards the center of the polyurea backing layer in an SPC plate owing to its in-contact interface, whose friction effect can be ignored. Therefore, when the intensity of air blast loads is large enough, stretching deformation instead of spalling occurs in a polyurea backing layer in SPC plates, as shown in the rightmost part of Figure 11.

### 4.5. Quantitative Analysis of Energy Absorption

The damage extent to a plate relies on the imparted impulse during the blast-resistant procedure, because the action time of the blast waves is very short [52]. Hence, the energy absorption for each layer of the tested plates can be calculated based on momentum and energy conservation laws. The details regarding the calculation procedure have been described in the literature [47]. For each layer in the polyurea-coated steel plates, the energy absorption was calculated and compared with that of the bare steel counterparts (from Ref. [47]).

**Figure 12:**
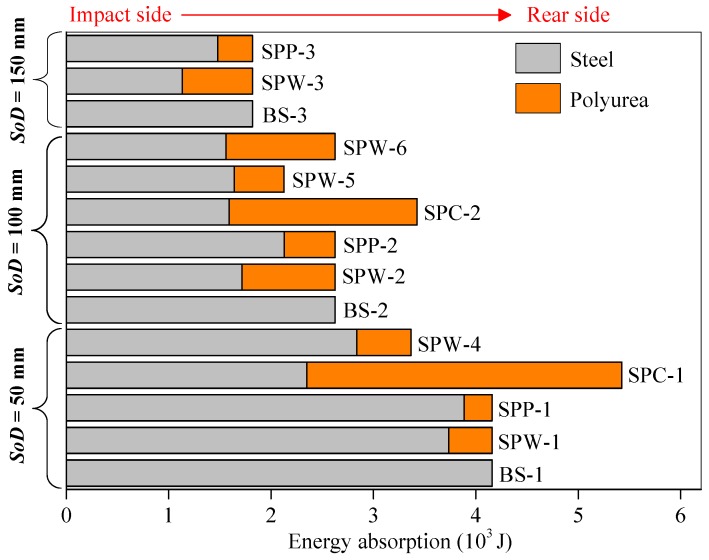
Influence of spraying strategy on the energy absorption of polyurea-coated steel plates.

As shown in Figure 12, the polyurea backing layer in an SPW plate absorbed a larger amount of kinetic energy than that in a SPP plate under the same stand-off distance conditions. This indicates that it is better to make use of the energy absorbing capability of the polyurea backing layer in whole-area sprayed polyurea-coated steel (SPW) plates due to the confinement of their boundaries. Figure 12 also shows that the energy absorption proportions of polyurea backing layers for both whole-area (SPW) and partial-area sprayed (SPP) plates are enhanced as the stand-off distance increases. This is mainly due to the fact that when the intensity of the air blast loading is relatively low, the polyurea backing layers have enough time to produce stretching deformation. This also suggests that the front steel layer in the direct-sprayed polyurea-coated steel plates, i.e., SPW and SPP plates, plays a dominant role in energy absorption. However, the polyurea backing layers in SPC plates absorb more kinetic energy than the front steel layers, as shown in Figure 12, mainly because the polyurea backing layers in SPC plates are inclined to produce stretching deformation and their energy absorbing capability can be more sufficiently utilized due to the existence of an in-contact interface. Moreover, the collision and friction between layers also absorbs a certain amount of energy in SPC plates.

By comparing the energy absorption in Figure 12 for SPW-5, SPW-2, and SPW-6, it can be concluded that the proportions of energy absorption for the polyurea backing layers in SPW plates increase as the relative thickness of polyurea the backing layer increases. This is mainly due to the following two aspects: (i) the energy absorption of the front steel layer is mainly related to its thickness and increases with increasing thickness and (ii) a relatively thicker polyurea backing layer absorbs more kinetic energy. Nevertheless, this conclusion is suitable for a certain range of relative thicknesses only, and excessive increasing of the thickness of the polyurea backing layer in a direct-sprayed polyurea-coated plate will deteriorate the blast-resistant performance of the front steel layers under an identical total areal density condition. Notably, in this study, the imparted initial impulses were considered identical for all the tested plates for the purpose of preliminary analysis.

## 5. Conclusions

The application of polyurea in protective structures suffering damage loads generated from intentional or unintentional explosions has grown rapidly owing to its potential improvement of overall structural resistance to explosions. Nevertheless, polyurea-coated structures manufactured with different spraying strategies have significantly different overall resistance performance when subjected to explosions; however, the underlying mechanisms are still not clear. This issue hinders the more widespread use of polyurea in the protective field, and is worth studying. The present work has aimed to illuminate the influence of spraying strategy on the resistance performance of polyurea-coated steel plates under localized air blast loading by conducting experiments, and an attempt was made to shed a light on the underlying mechanisms. The influence of spraying strategy, i.e., spraying area, spraying thickness, and spraying interface condition, on the performance of polyurea-coated steel plates resisting air blast loadings was evaluated experimentally. The differences in deformation and failure modes of plates resulting from different spraying strategies were identified. The underlying energy absorbing mechanisms of polyurea backing layers in different plates were elucidated. The efficiency of taking advantage of the energy absorbing capabilities of polyurea backing layers was quantitatively analyzed. The main conclusions drawn are as follows:

(1) The whole-area spraying of polyurea can make better use of the energy absorbing capabilities of polyurea backing layers compared with partial-area spraying, mainly due to the confined polyurea boundaries, resulting in large stretching deformation as well as spalling of the polyurea backing layer.

(2) A suitable increase in the spraying thickness of the polyurea backing layer in a certain range improves the air-blast-resistant performance of the whole-area sprayed polyurea-coated steel plates by keeping the same total areal density, but an excessive increase in spraying thickness decreases the overall performance.

(3) The in-contact interface can greatly reduce the damage of the front steel layer under intense air blast loading but has relatively lower efficiency in decreasing the deformation of the front steel layer in comparison with the spraying bonding interface at a relatively low intensity of air blasts.

(4) When maintaining an identical total areal density, polyurea-coated steel plates with whole-area spraying and in-contact backing of polyurea both exhibit superior performance to, and those with partial-area spraying of polyurea show inferior performance to, bare steel counterparts.

(5) For both whole-area and partial-area sprayed polyurea-coated steel plates, the dominant energy absorbing components are the front steel layers. However, the polyurea backing layer absorbs more kinetic energy than the front steel layer in an in-contact polyurea-coated steel plate.

## Figures and Tables

**Table 1:** Quasi-static material parameters of 304 stainless steel and LINE XS-350 polyurea.

Property	304 Stainless Steel	LINE XS-350 Polyurea
Density, *ρ*_s_ (g/cm^3^)	7.90	1.08
Elastic modulus, *E*_s_ (MPa)	2.05 × 10^5^	201
Quasi-static yield stress, *σ*_s_ (MPa)	310	—
Shore hardness (D)	—	60 ± 1
Tensile strength, *σ*_s_ (MPa)	736	22.39
Failure strain, *ε*_f_	0.41	1.63

**Table 2:** Schematic design of the three types of polyurea-coated steel plates with different spraying strategies. Legend: SPW, polyurea which is sprayed on the whole area of the back side of the steel plate; SPP, polyurea which is sprayed onto a partial area (central area) of the back side of the steel plate; SPC, polyurea in which the polyurea backing layer is in contact with the rear surface of the steel plate.

Specimen	Geometry	Spraying Position	Spraying Area	Interface Condition
SPW	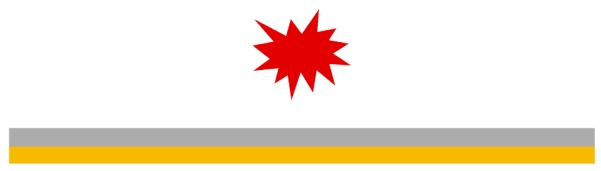	Back face	Whole area	Direct spraying
SPP	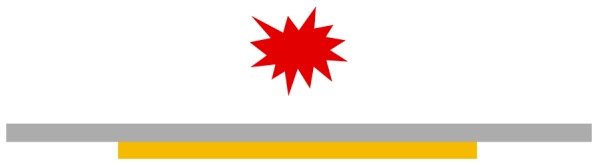	Back face	Partial area (central area)	Direct spraying
SPC	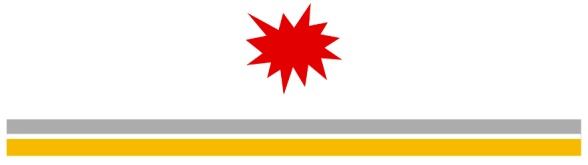	Back face	Whole area	In contact

■ Steel ■ Polyurea.

**Table 3:** Detailed information regarding the experimental results for polyurea-coated steel plates in the present study.

Case No.	Information about Tested Plates				Explosive		Experimental Results	
	*h*_s_ (mm)	*h*_p_ (mm)	*ρ*_A_ (kg/m^2^)	*W*_t_ (kg)	*SoD* (mm)	*W*_e_ (g)	*δ*_s_ (mm)	*M*_s_
SPW-1	1.38	3.1	14.2	2.72	50	55	Failed	Petalling
SPW-2	1.38	3.1	14.2	2.72	100	55	37.9	I
SPW-3	1.38	3.1	14.2	2.72	150	55	34.5	I
SPW-4	1.80	3.1	17.5	3.36	50	55	Failed	IIc
SPW-5	1.80	3.1	17.5	3.36	100	55	43.0	I
SPW-6	0.90	6.6	14.2	2.72	100	55	46.8	I
SPP-1	1.38	3.1	14.2	2.38	50	55	Failed	Petalling
SPP-2	1.38	3.1	14.2	2.38	100	55	Failed	IIc
SPP-3	1.38	3.1	14.2	2.38	150	55	47.5	I
SPC-1	1.38	3.1	14.2	2.72	50	55	Failed	Petalling
SPC-2	1.38	3.1	14.2	2.72	100	55	42.4	I

**Table 4:** Detailed information of the experimental results for bare steel plates [47].

Case No.	Information about Tested Plates				Explosive		Experimental Results	
	*h*_s_ (mm)	*h*_p_ (mm)	*ρ*_A_ (kg/m^2^)	*W*_t_ (kg)	*SoD* (mm)	*W*_e_ (g)	*δ*_s_ (mm)	*M*_s_
BS-1	1.80	-	14.2	2.72	50	55	Failed	Petalling
BS-2	1.80	-	14.2	2.72	100	55	55.0 ^a^	II *c
BS-3	1.80	-	14.2	2.72	150	55	39.5	I

^a^: maximum deflection of bulging region ignoring failure.

**Table 5:** Measurements of crevasses/holes of front steel layers in polyurea-coated steel plates and a bare steel plate, BS-1 (from Ref. [47]).

Case No.	*h*_s_ (mm)	*SoD* (mm)	*M*_s_	Petal Numbers	*d*_c_ (mm)
SPW-1	1.38	50	Petalling	7	200.9
SPW-4	1.80	50	IIc	/	33.4
SPP-1	1.38	50	Petalling	7	206.6
SPP-2	1.38	100	IIc	/	47.2
SPC-1	1.38	50	Petalling	4	102.8
BS-1	1.80	50	Petalling	7	165.9

**Table 6:** Measurements of spalling on the polyurea backing layers in the present experiments.

Case No.	Layer Thickness		Charge Information		Failure Mode	Area of Spalling	
	*h*_s_ (mm)	*h*_p_ (mm)	*SoD* (mm)	*W*_e_ (g)	*M*_s_	*A*_s_ (cm^2^)	*A*_s_/(*L*_e_·*B*_e_)
SPW-2	1.38	3.1	100	55	I	313.1	0.36
SPW-3	1.38	3.1	150	55	I	268.4	0.31
SPW-4	1.80	3.1	50	55	IIc	412.2	0.48
SPW-5	1.80	3.1	100	55	I	238.6	0.28
SPW-6	0.90	6.6	100	55	I	423.3	0.49
SPP-2	1.38	3.1	100	55	IIc	125.1	0.14
